# Patterned Array of Poly(ethylene glycol) Silane Monolayer for Label-Free Detection of Dengue

**DOI:** 10.3390/s16091365

**Published:** 2016-08-25

**Authors:** Nor Zida Rosly, Shahrul Ainliah Alang Ahmad, Jaafar Abdullah, Nor Azah Yusof

**Affiliations:** 1 Department of Chemistry, Faculty of Science, Universiti Putra Malaysia, Serdang 43400, Selangor, Malaysia; norzidarosly@gmail.com (N.Z.R.); jafar@upm.edu.my (J.A.); azahy@upm.edu.my (N.A.Y.); 2 Institute of Advanced Technology (ITMA), Universiti Putra Malaysia, Serdang 43400, Selangor, Malaysia

**Keywords:** UV-arrays, PEG silane monolayer, DNA dengue, gold nanoparticles

## Abstract

In the present study, the construction of arrays on silicon for naked-eye detection of DNA dengue was demonstrated. The array was created by exposing a polyethylene glycol (PEG) silane monolayer to 254 nm ultraviolet (UV) light through a photomask. Formation of the PEG silane monolayer and photomodifed surface properties was thoroughly characterized by using atomic force microscopy (AFM), X-ray photoelectron spectroscopy (XPS), and contact angle measurements. The results of XPS confirmed that irradiation of ultraviolet (UV) light generates an aldehyde functional group that offers conjugation sites of amino DNA probe for detection of a specific dengue virus target DNA. Employing a gold enhancement process after inducing the electrostatic interaction between positively charged gold nanoparticles and the negatively charged target DNA hybridized to the DNA capture probe allowed to visualize the array with naked eye. The developed arrays demonstrated excellent performance in diagnosis of dengue with a detection limit as low as 10 pM. The selectivity of DNA arrays was also examined using a single base mismatch and noncomplementary target DNA.

## 1. Introduction

Dengue fever, caused by the dengue virus (DENV), is transmitted through the bite of female mosquitoes, mainly of the species of Aedes aegypti and Aedes albopictus [1]. Dengue virus is a single-stranded RNA virus with approximately four antigenically serotypes (DEN-1, DEN-2, DEN-3, DEN-4) which causes a wide range of symptoms, such as fever, headache, severe joint and muscle pain. The symptoms normally appear in 3–14 days after the affective bite and primary infection of the virus will cause mild febrile disease, which potentially leads to life-threatening severe dengue (also known as dengue hemorrhagic fever) and dengue shock syndrom (DSS) should the symptoms are not treated [2,3].

The number of dengue fever cases reported in Malaysia has continued to soar every year since 2012 with alarming statistic in 2014. According to the Health Ministry of Malaysia, 336 people (an average of 28 a month) died from dengue in 2015, i.e., a 56.3% increment compared to 215 fatality cases in 2014 [4]. Reverse transcription-polymerase chain reaction (RT-PCR) amplification assay is one of the current laboratory tests used worldwide. RT-PCR in clinical serum samples shows that the limit of detection of twofold serial dilution of transcribed RNA varies from 6.0 × 10^2^ to 1.1 × 10^3^ GCE/mL, depending on sample time-point and DENV target. Despite their sensitivity, the tests are tedious, time consuming, involving expensive apparatus and likely to give false-positive reading due to cross contamination with dengue virus PCR product [5,6].

This shows the avid need for a fast and responsive diagnostic method, which is able to provide early detection for the associated symptoms to be treated accordingly, eventually reducing the number of fatality cases. A label-free DNA biosensor based on DNA hybridization has been identified as an alternative method in detecting the specific nuclei acid sequence more effectively due to its rapid, specific and selective detection as compared in real-time. The sensing device consists of single-stranded DNA molecules (ss-DNA) attached to a physiochemical transducer for converting DNA hybridization events into analytical signals which is independent of labels that interfere with the binding site of the analyte of interest. The incorporation of a label-free DNA biosensor with several types of transducers such as mass [7], electrical [8], piezoelectric [9], optical transducers [10] has been receiving considerable attention in various fields, ranging from health care to agriculture sectors. Currently, there is growing interest in developing label-free optical sensors, particularly fluorescent methods, for DNA detection. This technique, however, requires the use of expensive fluorophores and detection instruments. As a substitute for fluorescent technique, Mirkin and coworkers have demonstrated scanometric detection to analyze DNA array, inducing gold nanoparticle aggregation [11]. Recent works on scannometric detection microarray have been studied. Chung et al. employed enhancement of daunorubcin conjugated to gold nanoparticles (DNR-AuNPs) which specifically bind the double-stranded DNA on the microarray. This method successfully detected a hemagglutinin-subtyping DNA [12].

The incorporated fabrication into label-free biosensors studies leads to improved performance of diagnostics. Numerous studies have been carried out to design and fabricate biosensors. Traditionally, the fabrication was performed using electron-beam (e-beam) lithography [13], microcontact printing (µCP) [14], dip-pen nanolithography [15], or UV photolithography [16]. UV lithography is a successful technique which considers the availability of light sources and can be demonstrated on different types of surfaces. However, previous studies reported that the use of conventional UV light (e.g., deep UV) creates problems in fabricating surfaces. It is difficult for UV light to reach the surface due to absorption by oxygen molecules; therefore, a high vacuum system is needed [17].

To overcome the problem, some researchers have reported the use of UV-absorbing molecules such as 1-(2-nitrophenyl)-ethyl-5-trichlorosilylpentanoate [18] or triphenylsulfonium triflate [19] that can activate surface UV reactions. Xue and co-workers invented the use of a minimal UV exposure dosage (~1.35 J/cm^2^) under ambient conditions without using a high-vacuum system [20]. Alonso et al. studied site-selective immobilization of protein in which photoreactive groups such as the benzylthiocyanate group and trimethoxysilyl group were self-assembled on a silicon oxide surface. The terminal group was irradiated by UV light (254 nm) and isomerized to form an isothiocyanate. The isothiocyanate was immobilized with propylamine under formation of a thiourea bond [21]. In addition, Corn and colleagues demonstrated the construction of multicomponent DNA arrays on gold substrate for surface plasmon resonance (SPR) imaging studies of protein-DNA interaction. The multistep procedure involved the photopatterning of Fmoc-protected photolabile surface, leaving an array of bare gold. Prior to DNA attachment via bifunctional linker SSMCC, the surface was initially exposed to 11-mercaptoundecylamine (MUAM) solution. In order to create nonspecific adsorption of protein molecules, the background of arrays was subsequently functionalized with NHS ester derivative of poly(oligo(ethylene glycol) upon the removal of Fmoc protecting group [22].

**Figure 1:**
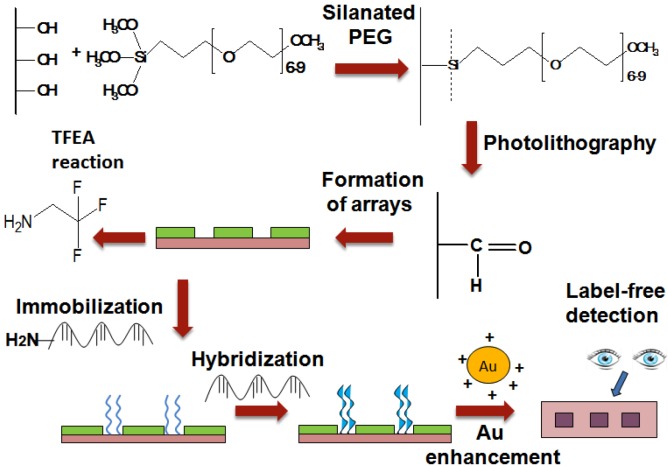
Schematic diagram of fabrication of UV-array polyethylene glycol (PEG) surface for label-free detection of dengue.

In this study, we presented the fabrication of UV-array of silane surfaces for the label-free dengue detection of synthetic dengue virus oligomers using the enhancement of gold nanoparticles (Figure 1). The arrays of PEG-silane were constructed by exposuse to 254 nm UV light followed by the immobilization of the amine DNA probe that later acts as a platform for the capture of the specific sequence of target DNA strands modeled on Dengue virus. The sequence of oligonucleotides for DNA probe and targets (as listed in Table 1) were chosen based on the relevant published work done by Yusof and coworkers, involving electrochemical sensing of silicon nanowires and gold nanoparticles [23]. The electrostatic binding of the positively charged gold nanoparticles and negatively charged hybridized DNA, following gold enhancement process results in the targeted spot being visible to the naked eye.

## 2. Experimental Details

### 2.1. Materials

All the synthethic oligonucleotides were purchased from First Base Laboratory Sdn Bhd, Selangor, Malaysia, with based sequences listed in Table 1. Silicon wafers, glass substrates and 1000-mesh copper grid were purchased from University Wafer, Boston, MA, USA (10 × 10 × 1 mm), Hirschmann Laborgerate (18 × 18 × 1 mm) and Agar Scientific, respectively. All chemicals used were obtained from R & M chemical (H_2_SO_4_, H_2_O_2_, HCl, and ethanol), Gelest (2-[methoxy(polyethyleneoxy)propyl] trimethoxysilane (PEG silane, *M*_W_ = 460−590)), Sigma-Aldrich (2-amino-1,1,1-trifluoroethane and hydrogen tetrachloroaurate (III) (HAuCl4)), Merck Milipore (sodium borohydrate (NaBH_4_)), Acros Organics (cetyltrimethylammonium bromide (CTAB)) and Nanoprobes, USA (GoldEnhance™-EM Formulation).

### 2.2. Characterizations

X-ray photoelectron spectroscopy measurement was performed with Kratos Axis Ultra (Model XSAM HS) using a monochromatic Al Kα X-ray source (energy 1486.6 eV) with pass energy 120 eV for wide scan and 40 eV for narrow scan of each element. All binding energy energies (BE) were calibrated by the BE (285.0 eV) of C1s, which gave BE values with an accuracy of 0.1 eV. The source operating with a base pressure the range of $10^{-8}$ m·bar to $10^{-10}$ m·bar. Data were processed using CasaXPS software. By using Voigt-type functions consists of both Gaussian and Lorentzian profiles (typically in the proportion 90% Gaussian to 10% Lorentzian), the peaks were fit with a linear background. Contact angle measurements were obtained with an VCA Optima Goniometer 3000 from AST (Billerica, MA, USA) equipped with a movable sample table and microliter syringe. Contact angles were measuremed from sessile drops by lowering a 5 μL drop from a syringe needle onto the surface. The resulting contact angle was averaged over the three time repeated measured data. Atomic force microscopy images were recorded by an Ambios Q-scope (Ambios Technology, Santa Cruz, CA, USA) SPM machine. All the images were obtained in air in contact mode with a silicon nitride cantilever. Transmission electron microscopy (TEM) and Field Scanning Scanning electron microscopy (FE-SEM) images were acquired with Hitachi H7100 (100 kV) and JEOL JSM 6400 microscope (15 kV), respectively. For TEM, the samples were prepared on standard copper TEM grids and dried for 15 h prior to analysis. For FE-SEM, the samples were fixed on a metal stub using carbon tape and then gold-coated using a sputter coater.

### 2.3. Formation of Poly(ethylene glycol) Silane Monolayer

First, substrates were cleaned by soaking them in a freshly prepared “piranha” solution of $H_{2}{\text{SO}}_{4}$:$H_{2}O_{2}$ (3:1) for 2 h, followed by rinsing with deionized $H_{2}O$ and drying under nitrogen atmosphere. The cleaned substrates were then immersed in a PEG silane solution that was prepared using 3 mM of 2-[methoxy(polyethyleneoxy)propyl] trimethoxysilane (PEG silane) by the dilution of deoxygenated toluene with 0.091 M of HCl. After 18 h immersion, the substrates were removed and washed in series starting with toluene, followed by twice with ethanol, then twice with water, and finally sonicated in ethanol for 2 min to remove any non-grafted material. The substrates were blown dry with nitrogen gas and immediately used or stored under ambient air conditions.

### 2.4. Preparation of Oligonucletides

Stock solution of all synthetic oligomers (100 µM) were prepared in (DI water, 18 MΩ/cm), which was obtained from a Milli-Q. The solution was kept frozen (−20 °C).

### 2.5. The Photolithography of PEGsilane and Formation of Arrays

**Figure 2:**
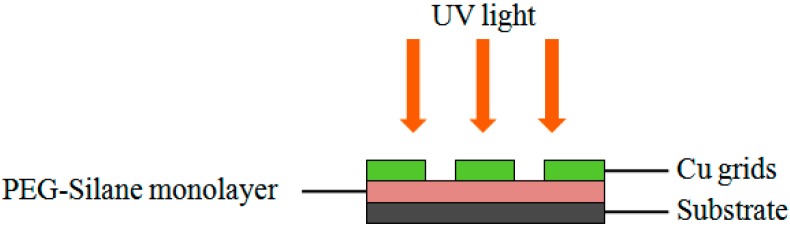
Schematic illustration of UV arrays.

The activation of surface functionality by the photocleavage of the PEG group was achieved by means of irradiation with a UV pen lamp (254 nm, 12.5 mW/cm² at 1.5 cm distance, Model 11SC-1) that was purchased from Spectronics (Westbury, NY, USA). For the construction of arrays, three grids were placed on the surface, with a distance between each grid of approximately 0.3 cm (Figure 2). The arrays were then used immediately for further functionalization as stated below.

### 2.6. TFEA Reaction

The reactivity of the irradiated surface was tested by reaction with 2-amino-1,1,1-trifluoroethane (TFEA). The samples were immersed in a solution of 10 mM of TFEA in ethanol for 3 h, removed, and then rinsed with ethanol and dried in a nitrogen atmosphere to prevent reactions to oxygen and water vapor. In all experiments, an exposure time of 5 min (1.35 J/cm²) was used.

### 2.7. Preparation of Positively Charged Gold Nanoparticles

A gold nanoparticles colloidal suspension was prepared according to previous work [12] by mixing 18.5 mL of distilled water, 0.5 mL of 10 mM sodium citrate, 0.5 mL of 10 mM hydrogen tetrachloroaurate (III) (HAuCl_4_), and 0.5 mL of 0.1 M sodium borohydrate (NaBH_4_). The solution was stirred for 3 h at room temperature. To create positively charged gold nanoparticle, the colloidal suspension was added with 0.5 mL of 10 mM cetyltrimethylammonium bromide (CTAB), a cationic surfactant. Finally, a phosphate-buffered silane (PBS) solution was added to the nanoparticle solution to create a similar hybridization.

### 2.8. DNA Probe Immobilization, Hybridization, and Label-Free Detection

The photo-modified surfaces were incubated with a 10 µM amine probe DNA for 1 h at room temperature with slight shaking. Then, they were rinsed three times with distilled water. The DNA-modified thin films were hybridized in a solution of target DNA at 37 °C for 2 h with slight shaking. After the samples were intensively washed with distilled water, a 2 mL solution containing the positively charged AuNPs was dropped onto the chip surface and incubated for 30 min at room temperature, followed by rinsing with PBS. The surface was reacted with a 2 mL gold enhancement solution for 3 min as outlined in Nanoprobes manual protocol followed by rinsing distilled water. To study the effect of various target concentrations on the DNA probe, the concentration of targets was varied from 10 pM to 1 µM. The gray levels of targeted spots were analysed by Photoshop. Each study was made in triplicate.

## 3. Results and Discussion

**Figure 3:**
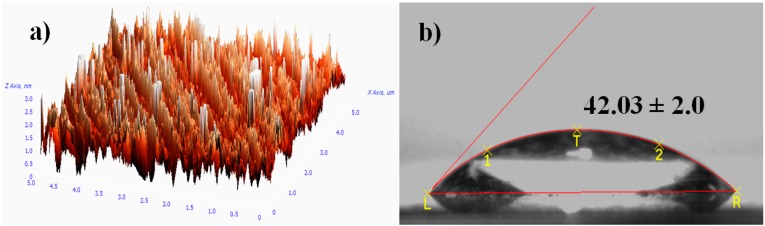
Analyses formation of PEG silane monolayer by (**a**) atomic force microscopy (AFM) image (size images: 7 × 7 µm²) (*Z*-range: 30 nm) and (**b**) optical image of contact angle.

Figure 3a shows the AFM image of the PEG silane monolayer. The image displays uniform growth of PEG silane molecules on the surface of the microscope slide. The successful coating of the monolayer on cleaned silicon is proposed by the observation of a low contact angle of around 42° (Figure 3b) which increases from 8° (cleaned substrate). The oxygen element in ether groups of PEGs was able to tetrahedrahedrally coordinate with water molecules via a hydrogen bond (H-bond acceptor), which leads to a lower contact angle and hydrophilic character [24].

**Figure 4:**
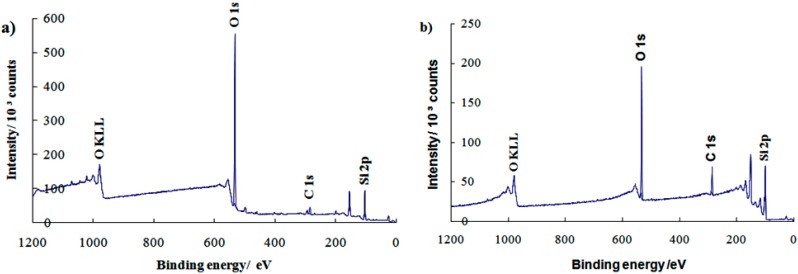
Wide scan X-ray photoelectron spectroscopy (XPS) spectra for (**a**) clean substrate and (**b**) PEG silane monolayer.

XPS analysis was further studied to determine the elemental composition and types of functional groups on the monolayer surfaces. Figure 4 shows the observation of sharp peaks around 532.0 eV (oxygen, O 1s), 285.0 eV (carbon, C 1s), and 104.0 eV (silicon, Si 2p) for both the (a) clean substrate and (b) PEG silane monolayer. The XPS data show that the small C 1s peak has increased in intensity with further treatment to form PEG silane. This phenomenon was due to the increased number of carbon atoms in the adsorption of alkylsilane molecules onto the substrate (Figure 3b). The presence of O KLL and other small peaks are due to the Auger peaks that contain many unwanted background electrons. The results of the study therefore indicate the presence of PEG silane molecules on the substrate surface.

**Figure 5:**
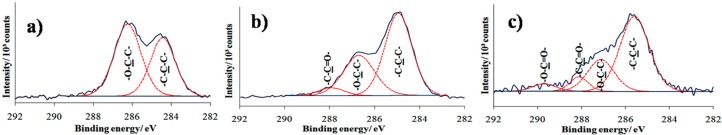
High resolution of C 1s XPS spectra of (**a**) PEG silane monolayer formation; (**b**) PEG silane monolayer being exposed to UV; and (**c**) 2-amino-1,1,1-trifluoroethane (TFEA) test on UV modified surfaces.

A high resolution spectrum of the C 1s region is shown in Figure 5. In Figure 5a, two distinct peaks are visible at 285.0 and 286.7 eV, attributed to C−C hydrocarbon and C−O ether carbon component, respectively. The ether peak was slightly larger than anticipated due to attenuation of the photoelectrons emitted from the underlying carbon atoms [24]. The post UV treatment in Figure 5b shows that the intensity peak at 286.7 eV decreases with an additional peak appearing at 288.2 eV, suggesting degradation of ethylene oxide and the formation of functional groups corresponding to aldehyde and carbonyl carbon groups. The result was consistent with previous studies reporting that photodegradation of the OEG chain leads to the reduction of ether component in the C 1s region and the presence of a new carbonyl component at 288.0 eV [25,26].

To study the reactivity of aldehyde functional groups, TFEA derivatization was performed. Analysis by contact angle showed an increased value in the contact angle from 46° to around 74°, suggesting the hydrophobicity of the surface that corresponds to the carbon atom bonded to the three fluorine atoms of TFEA. However, the value of the fluorinated terminated surface is lower than expected, likely due to steric hindrance that results in incomplete surface coverage CF_3_ of TFEA [27]. XPS analysis was further conducted to confirm the bond formation between the fluorinated amine of TFEA with aldehyde functional groups on modified surfaces. Previous work has reported that the chemical derivatization of aldehyde-terminated monolayer with TFEA resulted in the appearance of new peak in C 1s spectrum around binding energies 293.0 eV, corresponding to CF_3_ group [28]. However, Figure 5c shows the disappearance of CF_3_ peak for the sample after dipping to the TFEA solution. Nonetheless, a new peak is observed at binding energies at 289.7 eV that corresponds to carbonyl groups. The best description of this phenomenon is that the loss of perfluorinated organic molecules might be due to X-ray-induced damage. Researchers have reported that CF_3_ groups damage more rapidly than alkyl groups [29].

To enable the detection of negatively charged DNA by AuNP aggregation, nanoparticles with a net positive surface charge were prepared according to previously reported research [12]. The zeta potential of the AuNPs were determined at each stage of AuNP preparation. As shown in Table 2, the bare AuNPs initially carried a negative zeta potential due to the anionic citrate capping ligands. However, the zeta potential value became positive once the CTAB solution was introduced into the citrate solution of AuNPs. This positive potential remained after the colloidal suspension was mixed with PBS, indicating the CTAB ligands remain bound to the AuNPs.

**Figure 6:**
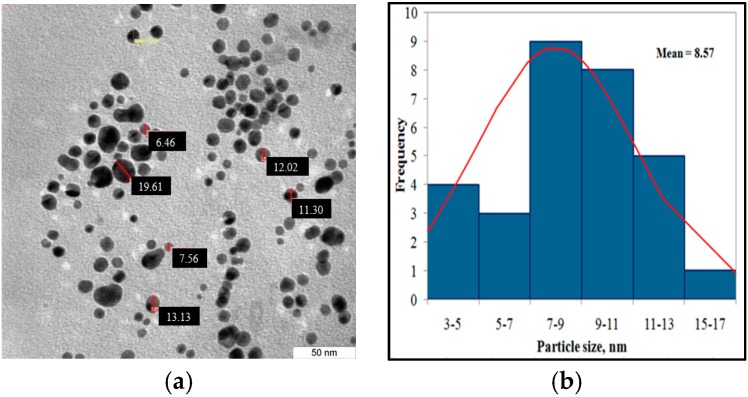
(**a**) A transmission electron microscopy (TEM) image of the cetyltrimethylammonium bromide (CTAB)-stabilized gold nanoparticles (scale bar: 50 nm); (**b**) Size distribution histogram.

These results are consistent with previous work [12] that showed the positively charged CTAB molecules replaced the citrate ligands on the AuNP surface. In other words, the stabilizing agent was exchanged from citric acid to CTAB due to the relatively high concentration of CTAB compared to the citric acid. The zeta potential slightly decreased due to the exchange of negatively charged phosphate ions in the PBS buffer for the positive ions from CTAB. The size of gold nanoparticles stabilized by CTAB micelles were measured by TEM. Figure 6a shows that the nanoparticles were found to be polydispersed with controlled spherical size. The nanoparticles exhibit a diameter (number-averaged) of 8.57 ± 4.7 nm. 

**Figure 7:**
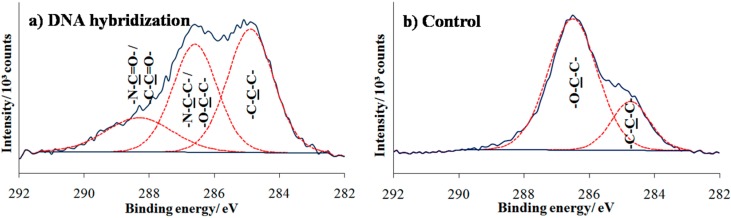
High resolution of C 1s XPS spectra for (**a**) DNA hybridization on photo-oxidized sample and (**b**) control sample.

To complete the preparation of the array for DNA detection, amino-functionalized probe DNA was immobilized onto the photo-oxidized surfaces. After complementary DNA target hybridization, the surfaces were analyzed by XPS as shown in Figure 7a. The result of C 1s spectrum shows some changes with the presence of a new peak at higher binding energy 288.2 eV that owes its existence to highly oxygenated carbon species (aldehyde or amide), indicating specific oligonucleotide adsorption to the UV-exposed areas. To verify that NH_2_-ssDNA was covalently linked to the oxidized area, control sample consisting of PEG-silane surface directly exposed to amino DNA probe solution which followed by target hybridization. As can be observed in Figure 7b, the two deconvoluted partial peaks at 285.0 and 286.7 eV that correspond to aliphatic hydrocarbon and C–O ether groups, respectively, remained unchanged and mostly similar to the peaks found in formation of the PEG silane monolayer (Figure 5a).

**Figure 8:**
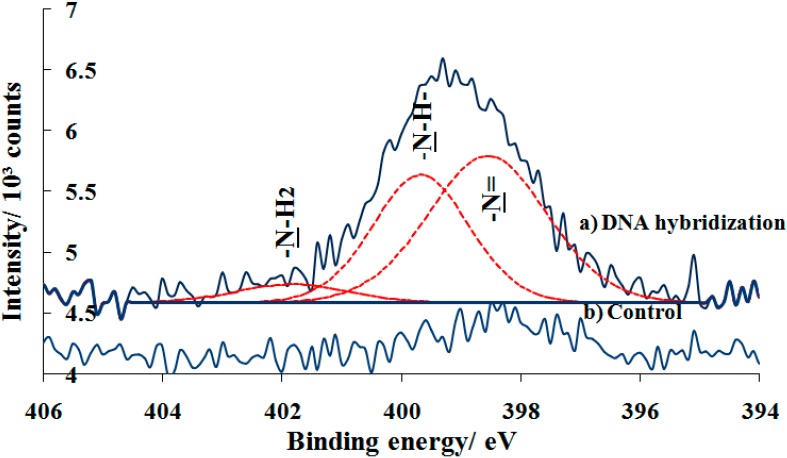
High resolution of N 1s XPS spectra for (**a**) DNA hybridization on UV modified sample and (**b**) control.

Further investigation on the specificity of hybridized DNA immobilization was shown in Figure 8a. The N 1s spectrum of DNA hybridized on photo-oxidized surface shows the broad and significant peak at 399.1 eV, indicating the presence of nitrogen-containing DNA bases and the covalent immobilization of amine-DNA probe on the surface. There peak deconvulated into three nitrogen components at approximately 398.6, 399.7 and 401.8 eV which are assigned to unsaturated chemical bond –N=, –NH– (secondary amine), and –NH_2_ (primary amine) cysteine and nucleic bases rings, respectively. Previous study of a different amide reported the presence of two peaks at 398.6 eV and 399.7 eV in the N 1s asymmetric signal due to the co-existance of free and hyrogen-bonded species. Additionally, the protonation of amide resulted in a chemical shift of the N 1s signal to higher binding energies due to the increase in the net positive charge in the nitrogen atom. It is reported that the shift of the N 1s signal to higher binding energies was related to the hybridization process [30]. Suprisingly, observing the N 1s spectrum for control sample, one can see that there was a small peak when hybridized DNA was tested on the PEG silane monolayer (Figure 8b). This is likely due to the challenge of forming uniform self-assembled PEG monolayer on substrate. Hence, this probably results in adventitious nitride or silazane (Si-N bonds) between Si from the substrate and nitrogen from amine-DNA probe/bases.

**Figure 9:**
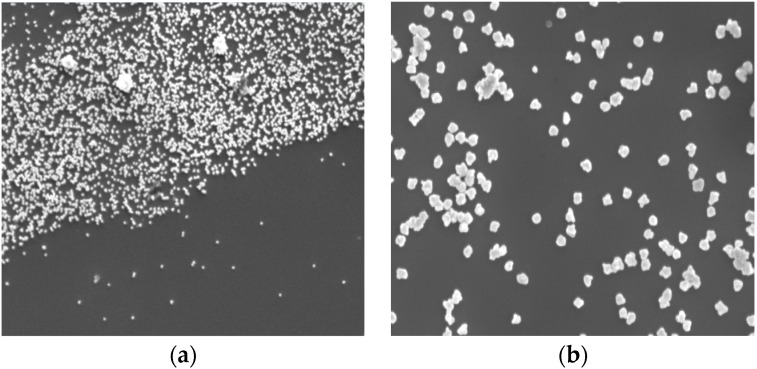
FE-SEM images of (**a**) targeted spot on array after gold enhancement process (scale bar = 4 µm) (**b**) Higher magnification of the spot (scale bar = 500 nm).

In order to demonstrate the colorimetric detection of DNA, the prepared array of immobilized DNA was exposed to a range of solutions containing differing samples of target DNA, followed by treatment with the CTAB-capped AuNPs and enhancement process. Successful hybridization of the target DNA to the array increased the overall surface negative charge, which then allows the capture of the positively charged AuNPs into aggregates that can be visually observed by employing enhancement solution. The solution is composed of gold ions and reducing agent in which the gold ions from the solution are catalytically deposited onto AuNPs, causing the nanoparticles to grow [11]. Figure 9a shows FE-SEM image of targeted spot of array after enhancement process. The border between the UV exposed and masked areas in which the gold nanoparticles were on exposed areas is clearly distinguished, indicating that the positively charged gold nanoparticles specifically bound to target-hybridized DNA. The magnified image (Figure 9b) that exhibits the size of gold nanoparticles was enlarged to be approximately 100 nm to 150 nm after enhancement process.

**Figure 10:**
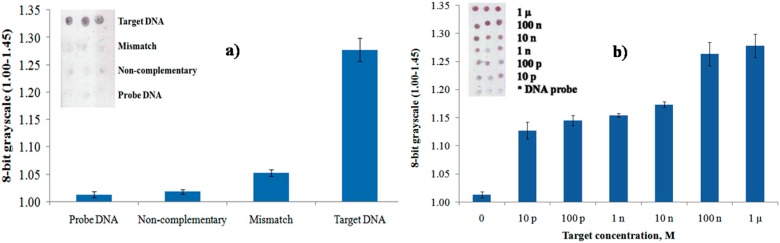
Graph of 8-bit grayscale values of 10 μM of probe DNA arrays respond towards (**a**) 1 μM of DNA solution of target complementary, mismatch and non-complementary (**b**) a range of complementary target concentrations (1 μM–10 pM), following nanoparticle treatment and enhancement process.

To study the selectivity of DNA probes, different DNA strands were used, including non-complementary, mismatch, and target DNA. Figure 10a shows grayscale levels for surface hybridization with different DNA strands (each at 1 μM) and DNA probe (10 μM) as a control after gold enhancement process. By using an 8-bit grayscale histogram in Photoshop, the images of each sample were analyzed. The result of hybridization with non-complementary DNA yielded a value of approximately 1.00, which was near the value for immobilization with the DNA probe. However, upon surface hybridization of probe DNA with mismatched DNA, the value increases and continues to increase with 1 µM complementary target DNA due to electrostatic interaction of the DNA strands and absorbed on the AuNPs. The data exhibits high selective discrimination between complementary and non-complementary sequences.

The responses of various target concentrations of DNA within a range of 1 μM–10 pM to the 10 μM of probe DNA were further studied by observing the gray levels of targeted spots which is comparable to the fluorescence and electrochemical methods [12,23]. Furthermore, based on the chart in Figure 10b, the intensity of the gray level increased with the increase of concentration of the target DNA within a range of 1 μM–10 pM, implying the larger amount of hybridized DNA are able to bind increasing amounts of cationic AuNPs. The gray level increased from the detection limit of 10 pM to the saturation point of 100 nM of the target DNA. The inset shows the optical scanner images of different target concentrations.

## 4. Conclusions

We have demonstrated the label-free detection of DNA strands modeled on Dengue virus gene sequence, though the use of a DNA-functionalized silicon-based array and nanoparticle aggregation upon capture of the complementary analyte DNA. These DNA-presenting arrays were produced from the photo-oxidation of PEG-terminated monolayers through a photolithographic mask using 254 nm UV light. The resultant aldehyde group has been selectively immobilized with an amine modified DNA probe for hybridization. The gold enhancement process after inducing the electrostatic interaction between positively charged gold nanoparticles and the negatively charged target DNA hybridized to the DNA capture probe was sufficiently sensitive that it enabled detection of as low as 10 pM of DNA by the naked eye with high specificity. The results indicate that the method used in the study offers sensitive and cost-effective diagnostic with simple visual of dengue detection which could be further developed for multiplex DNA arrays of early diagnosis in different serotypes of the dengue virus.

This successful demonstration of arrays for the label-free dengue detection is merely an example of many other potential applications, that can be developed using the method especially in future research of DNA arrays. This will be an opportunity to widen the scope and impact of nanotechnology such as utilizing of scanning near field photolithography [31] towards the development of nanofabricted devices. It would be far preferable to incorporate current and future technology with different sizes of nanoparticles to provide excellent sensitivity of measurement [12].

## Figures and Tables

**Table 1:** Sequences of oligonucleotides for DNA probe and targets.

Name of DNA Strand	DNA Sequence
Amine probe DNA	5′N$H_{2}$-AAC AGC ATA TTG ACG CTG GGA AAG ACC-3′
Target DNA	5′-GGT CTC TCC CAG CGT CAA TAT GCT GTT-3′
Non-complementary	5′-TTC TGT GTT AGT ATC TGG GCC ATG TCC-3′
Mismatch	5′-TTC AGG CTT CGA ATG TGG CGC ATG ACC-3′

**Table 2:** Zeta potential values with gold nanoparticles in different solutions.

Solution	Zeta Potential (mV)
Gold nanoparticles	−14.4 ± 1.7
Gold nanoparticles + CTAB	18.3 ± 3.1
Gold nanoparticles + CTAB + PBS	13.2 ± 2.3
